# COVID-19 and Delayed Cerebral Ischemia—More in Common Than First Meets the Eye

**DOI:** 10.3390/jcm10122646

**Published:** 2021-06-16

**Authors:** Pervinder Bhogal, Levansri Makalanda, Ameer E. Hassan, Dave Fiorella, Tommy Andersson, Muhammad Ahmad, Hansjörg Bäzner, Ounali Jaffer, Hans Henkes

**Affiliations:** 1 Department of Interventional Neuroradiology, The Royal London Hospital, Barts NHS Trust, Whitechapel Road, Whitechapel, London E1 1BB, UK; Levansri.makalanda@nhs.net; 2 Departments of Neurology and Radiology, University of Texas Rio Grande Valley, Harlingen, TX 78550, USA; ameerehassan@gmail.com; 3 Department of Neurosurgery, Stony Brook University Hospital, Stony Brook, NY 11794, USA; David.Fiorella@stonybrookmedicine.edu; 4 Department of Interventional Neuroradiology, The Karolinska University Hospital, 171 76 Stockholm, Sweden; tommy.andersson@karolinska.se; 5 4C Pharma Solutions LLC, Piscataway, NJ 08854, USA; mahmad@4cpharma.com; 6 Department of Neurology, Klinikum Stuttgart, 70174 Stuttgart, Germany; h.baezner@klinikum-stuttgart.de; 7 Department of Interventional Radiology, The Royal London Hospital, Barts NHS Trust, Whitechapel Road, Whitechapel, London E1 1BB, UK; ounali.jaffer@nhs.net; 8 Department of Neuroradiology, Klinikum Stuttgart, 70174 Stuttgart, Germany; hhhenkes@aol.com

**Keywords:** COVID-19, microthrombosis, delayed cerebral ischemia, subarachnoid haemorrhage

## Abstract

Since the arrival of the global COVID-19 pandemic scientists around the world have been working to understand the pathological mechanisms resulting from infection. There has gradually been an understanding that COVID-19 triggers a widespread endotheliopathy and that this can result in a widespread thrombosis and in particular a microthrombosis. The mechanisms involved in the microthrombosis are not confined to infection and there is evidence that patients with aneurysmal sub-arachnoid haemorrhage (SAH) also suffer from an endotheliopathy and microthrombosis. In this article we attempt to shed light on similarities in the underlying processes involved in both diseases and suggest potential treatment options.

## 1. Introduction

Since the beginning of 2020, COVID-19 has spread rampantly globally, with over 50 million confirmed cases and deaths exceeding one million. In unison with monumental efforts to manage the clinical caseload, there has been a worldwide effort to understand the virus and its effects on the body, and to develop appropriate management strategies. Additionally, respiratory failure coagulopathies are a devastating central mechanism of COVID-19. Interventional neuroradiology has been on the frontline with this disease in clinical care and in managing patients presenting with acute large vessel occlusions. Ostensibly, the interventional neuroradiologist and their knowledge base may not extend much beyond this clinical role. However, diseases with which we are familiar—subarachnoid hemorrhage and delayed cerebral ischemia (DCI)—appear to have more in common with COVID-19 than previously appreciated. 

This article attempts to highlight several commonalities between DCI and COVID-19, namely, endotheliopathy, derangement in the von Willebrand Factor (vWF)-platelet axis, and microthrombosis. We also suggest a potential mechanism for the rare occurrences of thrombosis seen after vaccination. We suggest potential therapies that may serve as treatment strategies for both diseases.

## 2. Overview of the vWF, ADAMTS13, Platelets, and Thromboinflammation

**Figure 1:**
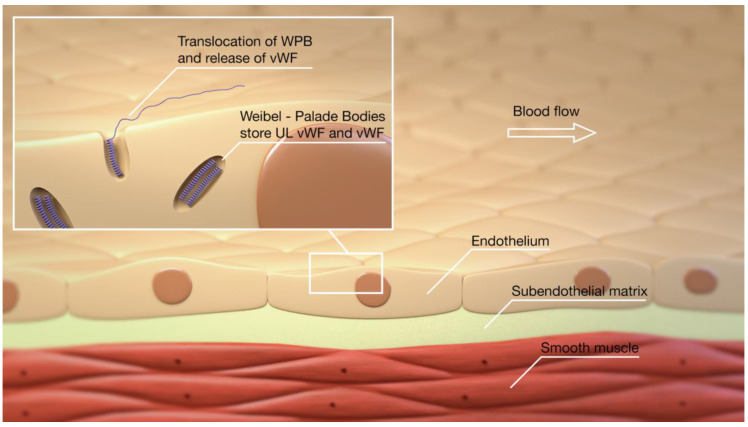
VWF is stored in the WPB and, under stimulation of the endothelium, is released into the blood stream.

**Figure 2:**
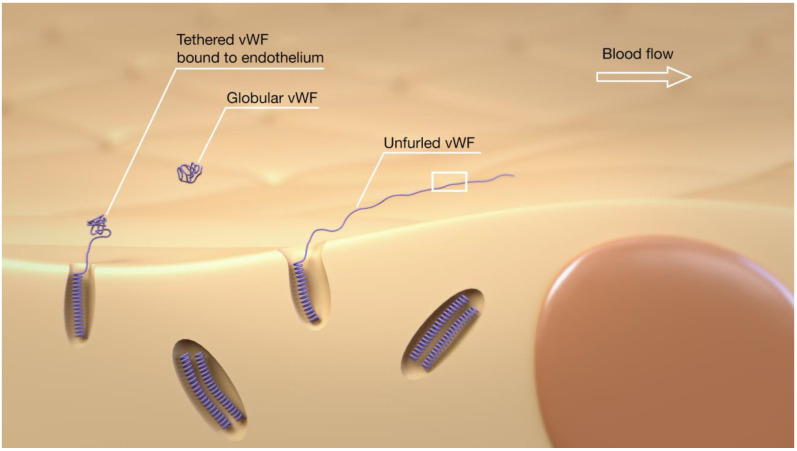
Untethered vWF is globular, whereas vWF tethered to the endothelium will unfurl as a consequence of the forces generated by the blood flow. The unfurling of the vWF molecule will expose sites involved in self-binding, platelet adherence, and for cleavage by the enzyme ADAMTS13.

**Figure 3:**
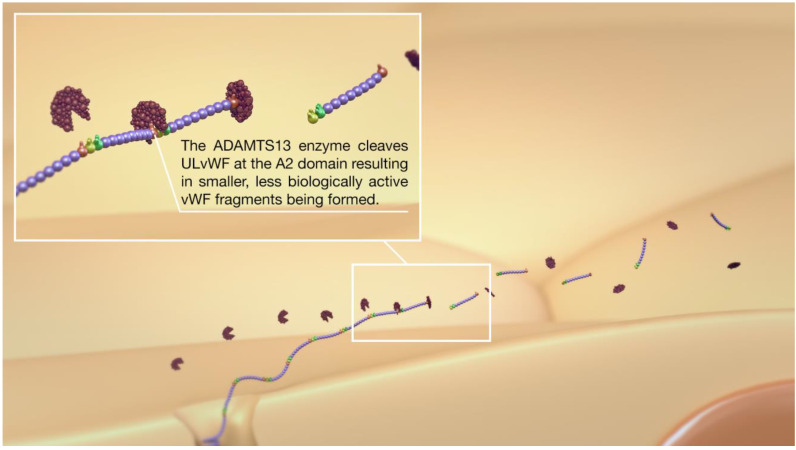
After unfurling, several important domains are exposed. The A2 domain allows for cleavage of ULvWF into smaller, less biologically active vWF molecules, and hence, hemostasis is regulated.

To understand the potential similarities between DCI and COVID-19, it is essential first to understand the critical role of vWF in normal hemostasis. Von Willebrand Factor is produced exclusively by endothelial cells (ECs) and megakaryocytes. During its biosynthesis, vWF undergoes significant dimerization and multimerization during production and post-translational processing. It is stored within the Weibel–Palade bodies (WPB) of the endothelial cells or megakaryocytes’ alpha granules (Figure 1). Although restricted to ECs, there are differences in the synthesis of vWF within the body’s different vascular beds, with the small vessels of the lung and brain expressing higher levels of vWF than similar-sized vessels of the liver or kidney [1]. The stored vWF multimers are enriched into Ultra-Large vWF (ULvWF) and prothrombotic forms released upon stimulation of the endothelial cells (Figure 2) and platelets. Von Willebrand Factor is secreted into the plasma in this ultra-large form that consists of several hundred vWF monomers. The biological function of vWF depends mainly on the size of its multimers [2,3,4,5]. The larger multimers are more likely to bind platelets and promote platelet adhesion in circulating blood; thus, ULvWF multimers are the most biologically active, inducing platelet aggregation [6]. Given the highly pro-thrombotic potential of ULvWF, mechanisms exist to degrade the ULvWF into smaller vWF forms, which are active in hemostasis but are no longer prothrombotic, to maintain a delicate hemostatic balance. This is achieved via the vWF-cleaving protease ADAMTS13 (A Disintegrin-like and Metalloprotease with Thrombospondin type 1-motif 13). The ADAMTS13 enzyme targets the A2 domain of the vWF and cleaves the hyperactive ULvWF multimers into smaller and less biologically active forms (Figure 3). Two contrasting disorders exemplify disturbances in this pathway—thrombotic thrombocytopenic purpura, where either an inherited deficiency of ADAMTS13 or the development of autoantibodies to ADAMTS13 results in a small vessel and arterial thrombotic phenotype, or type 2 von Willebrand’s Disease, in which large vWF multimers are defective or absent, resulting in a lifelong bleeding disorder.

The endothelial glycocalyx is a complex layer of proteins and carbohydrates that covers the vascular endothelium, both arterial and venous, which is composed principally of plasma proteins, proteoglycans, and glycoproteins along with their bound glycosaminoglycan (GAG) chains. Core proteoglycans, such as the syndecans and glypicans, support binding sites for GAGs that include heparan sulfate and chondroitin sulfate, which in turn protrude into the vascular lumen. The glycocalyx has many physiological functions that include the vascular anticoagulation and mechano-transduction of shear stress. The net negative charge of the glycocalyx, generated by the anionic oligosaccharides from which it is composed, results in an electrostatic repulsion against platelets and red blood cells, whilst the depth of the glycocalyx helps to physically separate blood cells from the endothelial surface. To date, there is limited literature available on the interaction between vWF and the glycocalyx; however, some studies suggest that the glycocalyx plays a role in tethering secreted and circulating vWF to the endothelium [7,8].

**Figure 4:**
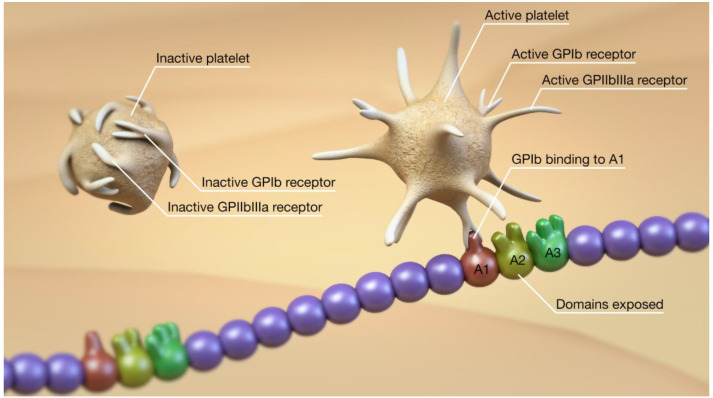
Unfurling of the vWF also exposes the A1 domain that represents the binding site for platelets to vWF via the platelet GPIb receptor.

Von Willebrand Factor has three well-recognized hemostatic functions, which mediate platelet–platelet interactions, platelet–collagen interactions, and act as a carrier for Factor VIII. Research also suggests that it plays a role in inflammation, angiogenesis, and smooth muscle cell proliferation. Upon secretion from the ECs, the ULvWF is in its globular form (Figure 2), with a proportion of the ULvWF released remaining bound to the endothelium [9,10]. Under hemodynamic shear stress, ULvWF unfolds, and binding sites for platelets (Figure 4), self-association, and adhesion to the vessel wall are exposed. Previous studies have shown that ULvWF molecules can self-associate into long “strings” in the direction of flow, both arterial and venous, that bind to platelets and leukocytes and adhere to the endothelium [10,11,12]. These strings can be remarkably long and have been seen to reach up to 1 mm in length [10]. The ULvWF multimers released from the WPBs have lower shear stress for unfolding. They, therefore, may represent the initiating molecules for this self-assembly process, which leads to hyper-adhesive strings capturing platelets. 

**Figure 5:**
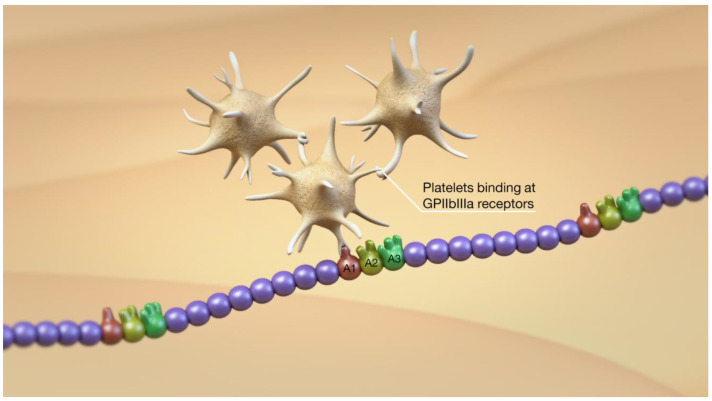
After binding of the platelet to vWF via the GPIb receptor, there is conformational change in the GPIIbIIIa receptor and platelet–platelet binding, resulting in the beginnings of platelet plug formation.

**Figure 6:**
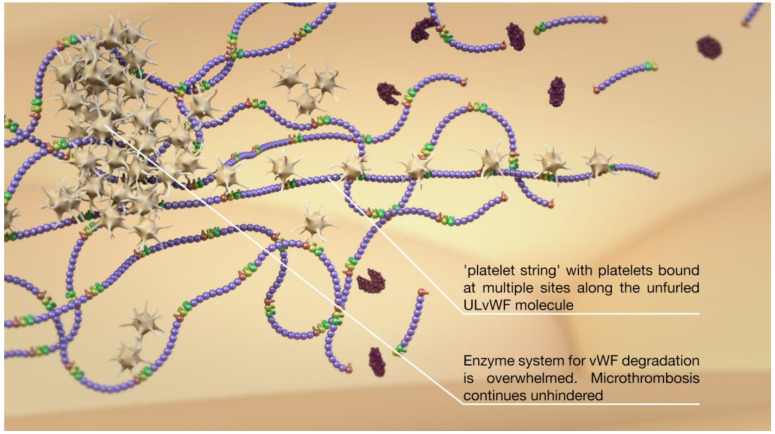
Multiple ULvWF molecules can intertwine with one another and bind platelets, resulting in platelet strings.

**Figure 7:**
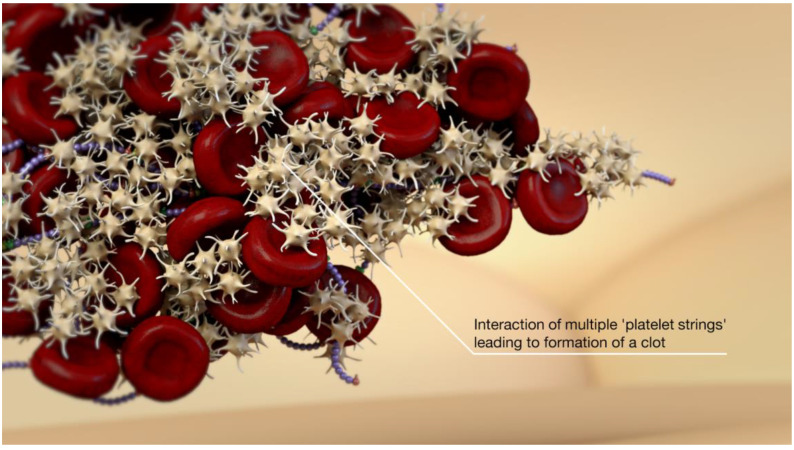
Left unchecked, there is thrombosis formation.

The binding of platelets to the ULvWF occurs via the GPIb receptor at the A1 domain (Figure 4). This receptor is usually not exposed when the ULvWF is in its globular form and cannot bind to platelets. Once ULvWF unfurls, secondary to shear stress, the receptor is exposed and binds very strongly to platelets. Platelets bound to the ULvWF are activated with conformational changes to the GPIIbIIIa receptor on their surface, allowing platelet–platelet binding (Figure 5) and the further formation of a platelet plug (Figure 6 and Figure 7). In addition to the A1 domain becoming exposed, exposure of the A2 domain during the unfurling of ULvWF allows the cleavage of ULvWF into the smaller, less biologically active molecules, as mentioned earlier. These two mechanisms need to work in balance to prevent prothrombotic or prohemorrhagic conditions, as mentioned earlier. Both platelet-decorated and platelet-free vWF strings are cleaved by ADAMTS13 at similar rates, suggesting that platelet binding has little influence on proteolysis [12]. If left unchecked, the release of ULvWF can result in microthrombosis.

Given that vWF and ULvWF are produced and released by endothelial cells, levels of vWF have been used as an indicator of an underlying endothelial injury, termed endotheliopathy. Therefore, the plasma level of vWF can be used as a marker of endothelial activation, vascular inflammation, and endotheliopathy. Raised levels of vWF are associated with acute respiratory distress syndrome and sepsis, and correlate independently to mortality [13,14], with evidence that the vWF axis is involved in delayed cerebral ischemia (DCI) secondary to aneurysmal subarachnoid hemorrhage (SAH) as well as COVID-19.

## 3. Evidence of Abnormalities in SAH and DCI 

In patients with SAH, who do not immediately perish, DCI represents a potentially treatable cause of a poor outcome. Large vessel vasospasm was thought to be the principal cause of this poor outcome. However, this view has been challenged recently, and there has been a more critical investigation into alternative reasons for the poor outcomes seen in some patients. One avenue of research has focused on developing microvascular thrombosis and microthrombi forming within the small arterioles, capillaries, and venules of the brain. There is a growing body of evidence, both in vivo and in humans, to suggest that microthrombosis plays a vital role in DCI. Several decades ago, microvascular thrombi were seen in the brains of patients who had died of aneurysmal SAH [15,16,17,18]. Stein et al. performed a histological analysis of the brains of 29 patients who had died from aneurysmal SAH [19]. The authors found a strong correlation between the extent of microvascular thrombi and the presence of ischemic infarctions. Microvascular thrombosis was most prevalent in brain areas with maximal neuronal necrosis, and they observed a bimodal peak in the development of microthrombosis formation with one peak in the first three days and a second peak between days 7 and 15. The results of this study suggest that microthrombosis plays a vital role in the development of DCI. 

A large number of animal studies have shown microthrombosis developing secondary to induced SAH. In rodent models of SAH using autologous blood infusion, microthrombosis and occluded blood vessels occurred throughout the brain within two days post-SAH [20] and could be observed for up to seven days post-ictus [21]. Microthrombosis has also been observed in puncture models of SAH [22,23,24]; however, as the puncture itself could act as a source for microthrombi, the autologous blood transfusion models may provide greater insight into the role of microthrombosis.

Von Willebrand Factor and ADAMTS13 have also been coplayers in microthrombosis. Muroi et al. investigated the role of ADAMTS13 in SAH in a mouse model [25]. They showed that the systemic administration of ADAMTS13 achieved significant amelioration of microthrombosis and neurologic performance with a reduction in the amount of apoptotic and degenerative neurons. Remarkably, ADAMTS13 had no significant effect on vasospasm, and the administration of ADAMTS13 neither increased the amount of subarachnoid blood nor prolonged the bleeding time.

Vergouwen et al. used a prechiasmatic injection model of SAH to assess the potential therapeutic benefit of ADAMTS13 treatment on SAH-induced microthrombosis formation [26]. They used recombinant human ADAMTS13 (rADAMTS13) delivered intravenously to wild-type and ADAMTS13 knockout mice following SAH induction. They showed a trend towards a reduction in microthrombosis in wild-type mice and a significant reduction in microthrombosis in the knockout mice.

Wan et al. sought to determine the role of vWF and ADAMTS13 in SAH [27]. They used a prechiasmatic blood injection model in wild-type controls, ADAMTS13 knockout, and vWF knockout, and wild-type mice were given recombinant rADAMTS13. They showed that the vWF knockout mice and those treated with rADAMTS13 had significantly less neuronal injury than the wild-type controls (64 vs. 185 neurons, *p* < 0.01; 45 vs. 185 neurons, *p* < 0.001, respectively). There was also a trend towards reduced neuronal injury in the ADAMTS13 knockout mice. The authors suggest that this study demonstrates the critical role vWF plays in early brain injury after SAH. This study was not designed to observe the development of microthrombosis, which typically occurs at 48 h post-ictus in mice; however, taken together with previous studies, it suggests that the vWF-ADAMTS13 axis is involved in DCI.

In a pilot study of 40 consecutive patients, Kumar et al. assessed the ADAMTS13 and vWF levels on days 0, 1, 3, 5, 7, and 10 postictus compared to healthy controls [28]. They showed that vWF activity and vWF antigen (the routine marker for vWF) were significantly higher in SAH patients compared to controls at each time-point (*p* < 0.0001). Similarly, there was a marked reduction in plasma ADAMTS13 activity in the SAH patients compared to controls (*p* < 0.0001), as was the ratio of plasma ADAMTS13 and vWF antigen (*p* < 0.0001). 

Similar results were shown previously by Vergouwen et al., with more marked decreases in ADAMTS13 seen in patients who developed DCI than in those that did not (*p* = 0.0001) [29]. They also observed more pronounced increases in vWF antigen and the vWF-precursor, vWF propeptide (*p* = 0.02 and 0.004, respectively). 

Further studies have shown a derangement in the glycocalyx lining of the endothelium in patients with SAH [30], consistent with other groups’ findings suggesting a significant insult to the vascular endothelium post-SAH. Collectively, these observations indicate that the vWF–ADAMTS13 axis plays an essential role in DCI development.

## 4. Evidence of Abnormalities in COVID-19

There is an increasing recognition that COVID-19 causes endotheliopathy [31,32,33,34,35,36], and widespread microthrombosis is seen using contrast-enhanced ultrasound [37,38]. Autopsy studies have shown evidence of extensive, potentially fatal endotheliopathy. Varga et al. demonstrated a widespread endotheliopathy affecting the pulmonary, renal, gastrointestinal, and hepatic vessels on post mortem examinations of three patients with COVID-19 [35]. In one of the cases, the authors reported that “most of the small vessels appeared congested”. In another case, the patient died from bowel ischemia with evidence of underlying endotheliopathy. 

In a case series of COVID-19 pulmonary autopsies, it was revealed that, alongside diffuse alveolar damage, numerous localized platelet-rich microthrombi and foci of hemorrhage were present in the lungs [39]. The authors posited pulmonary-localized thrombotic microangiopathy as a key to the pathogenesis of COVID-19, with others also suggesting microthrombosis as a critical driver in the disease process [40]. In the case series of Carsena et al., microthrombi in vessels <1 mm in diameter were seen in 87% of cases [41].

Multiple studies also revealed microthrombosis on brain biopsy and post mortem studies [42,43,44,45]. Consistent with the fact that COVID-19 causes a widespread endotheliopathy is the evidence of marked rises in vWF. The earliest report of a substantial elevation of vWF was that by Escher et al., who reported an increase in vWF of over 500% [34]. Subsequently, Goshua et al. reported marked elevations in plasma vWF concentrations in COVID-19 patients with increased levels associated with disease severity. Mean vWF antigen levels of those admitted to the ICU were 565 ± 199% vs. 278 ± 133% of non-ICU patients (*p* < 0.0001). Other groups have also shown marked elevations of vWF in patients with COVID-19 [46,47,48]. Rauch et al. looked at the clinical course of patients with COVID-19 in relationship to their vWF levels on admission [49]. They showed that patients with the initial highest vWF levels required more oxygen support.

In contrast, the small cohort (*n* = 10) attended the emergency department presenting normal vWF levels and required neither hospitalization nor supplementary oxygen. Ladikou et al. also observed increased vWF antigen levels in ICU COVID-19 patients, positively correlating with the patients’ ages [36]. This group also showed reduced levels of ADAMTS13 (49.7%), and it was estimated that this reduction in plasma ADAMTS13 levels was secondary to the excess release of vWF and consumption. South et al. measured a six-fold increase in UL-vWF levels of COVID-19 patients compared to healthy controls, possibly due to an imbalance in IL-6-driven release and reduced ADAMTS13 [50]. More recently, Philippe et al. [51] showed a strong association between ULvWF and outcome. In the most extensive study to date (*n* = 208), the authors showed a marked difference in the vWF:Ag levels between critical (507%) and noncritical (288%) patients, which was highly significant (*p* < 0.0001). On further analysis, the authors showed that the ROC for vWF:Ag was the most predictive for mortality with an AUC of 0.92 (95% CI 0.88–0.96) and a cut-off level of vWF:Ag (423%), able to predict mortality in univariate and multivariate modeling, the Kaplan–Meier estimator, and the Cox proportional hazard. Taken together, these studies suggest that there is a marked abnormality in the ratio of ULvWF and the regulatory enzyme ADAMTS13 that tips the system into a markedly prothrombotic state. Huisman et al. [52] were the first to show a mean vWF:ADAMTS13 ratio of 8.5 (normal 0.5–2) from 12 patients admitted to the ICU. Subsequently, Mancini et al. [53] demonstrated similar findings with an elevated vWF:Ag to the ADAMTS13 activity ratio, which was strongly associated with disease severity with the worst ratio, 8.3, seen in the patients that required high-intensity care (intubation and mechanical ventilation) compared to those requiring low-intensity care, 3.42 (*p* < 0.001). Another disease caused by an abnormal vWF:ADAMTS13 ratio is thrombotic thrombocytopenic purpura (TTP). In the acquired form of TTP, a circulating autoantibody that inhibits the function of ADAMTS13 is produced; hence, vWF platelet microthrombi formation can occur unhindered due to a lack of functioning ADAMTS13. In a sense, acquired TTP is caused by an abnormal ratio of vWF, which may be released in normal quantities, and nonfunctioning ADAMTS13, whereas in COVID-19, there is a massive release of ULvWF and regular or reduced levels of ADAMTS13, secondary to consumption, that result in microthrombosis. Doevelaar et al. [54] recently published the results of their series of 75 patients in whom a vWF multimer gel analysis was performed. The other groups mentioned above showed an increase in the vWF:Ag levels and a derangement in the vWF:ADAMTS13 ratio. They also showed that a vWF multimer pattern that resembled TTP with a smeary triplet pattern, an indicator of ADAMTS13 dysfunction, was seen in 39% of cases. The authors state “COVID-19 is associated with a substantial increase in von Willebrand factor levels, which can exceed the ADAMTS13 processing capacity resulting in the formation of large von Willebrand factor multimers indistinguishable from thrombotic thrombocytopenic purpura”. 

A summary of the core similarities seen between COVID-19 and SAH is shown in Table 1.

## 5. Potential Treatment Strategies 

Overall, there is growing evidence for underlying endotheliopathies in both DCI and COVID-19, expressed by consistent rises in vWF. The significant increases in vWF could result in a consumptive effect on the available ADAMTS13 levels and cause a prothrombotic state. This implies potential benefits of early interventions targeting the vWF-ADAMTS13 axis in both diseases. 

### 5.1. GPIb Receptor Antagonists

As mentioned earlier, platelets bind to the GPIb receptor, located on the A1 domain, on ULvWF. Two available drugs are inhibiting this interaction—caplacizumab (Sanofi) and anfibatide (Lees Pharma). Caplacizumab targets the A1 domain of the ULvWF, inhibiting the interaction between ULvWF and platelets, hence preventing platelet adhesion and microvascular thrombosis. Two trials investigated caplacizumab to treat thrombotic thrombocytopenic purpura (TTP): TITAN and HERCULES [55,56]. Both trials met their primary end-points along with good safety profiles (reduced numbers of thromboembolic adverse events). In total, 11.4% of caplacizumab-treated patients and 43.2% of placebo-treated patients experienced one or more major thromboembolic events, an exacerbation, or died in the TITAN study. Bleeding events were generally mild or moderate, with epistaxis and gingival bleeding in TITAN. Not surprisingly, bleeding risk was higher in the caplacizumab group (54% of patients vs. 38% in the placebo arm). Of the 101 bleeding-related adverse events, 83% (*n* = 84) were mild (transient and not requiring more than minimal intervention), and 14% were moderate (alleviated by therapeutic intervention and causing discomfort, but without significant or permanent risk of harm). In the HERCULES cohort, caplacizumab 10 mg significantly reduced the time-to-confirmed normalization of the platelet count by 39% compared with placebo (median 2.97 vs. 4.79 days; *p* = 0.005), with complete remission observed more frequently in the caplacizumab arm (81% vs. 46%).

Anfibatide, a snake-venom-derived GPIb inhibitor, is an alternative agent and is currently undergoing investigation for TTP use. For caplacizumab, and in all likelihood anfibatide too, plasma-derived von Willebrand factor concentrate can act as an antidote. Both of these agents would represent potential treatment options for patients with SAH; however, given that they inhibit binding platelets to vWF and platelet activation, their use should be considered after the ruptured aneurysm is secured. Inhibiting the initial stage of platelet–vWF binding would be advantageous since ULvWF platelet strings are sufficient to occlude the microvasculature alone without the need for platelet–platelet binding. Several authors have now suggested that the use of caplacizumab or anfibatide [51,54,57] may represent a potential treatment option for patients with COVID-19. We believe that the use of GP1b receptor antagonists would be feasible and should be considered for patients with vWF:Ag levels of over 200% and vWF:ADAMTS13 ratios of ≥4 based on the existing literature. 

### 5.2. N-Acetyl Cysteine

Intravenous N-Acetyl Cysteine (NAC), which has been shown to reduce the size of vWF multimers, represents another attractive treatment option and has a long history and familiarity amongst clinicians. In patients with elevated vWF levels, but normal ADAMTS13 levels, IV NAC could aid in the degradation of ULvWF; in patients with more markedly deranged vWF:Ag and ADAMTS13 levels, it could be used in conjunction with GPIIbIIIa blockers, as this combination has previously been shown to significantly impair clot formation [58]. When using these drugs, it is essential to note that high vWF levels may impede the effect of GPIIbIIIa antagonists, and eptifibatide may offer advantages over tirofiban in this regard [59]. It is important to note that NAC should be used as part of a preventative strategy and, therefore, would need to be started early in patients at high risk of disease progression, since NAC will not degrade any microthrombi already formed. This strategy could also be readily applied to patients with SAH with IV NAC instigated on admission and the instigation of GPIIbIIIa receptor blockade used in select cases following the aneurysm’s securing. Similarly, the fact that it is the GPIIbIIIa receptor that is activated by the binding of platelets to the GPIb receptor could explain why aspirin fails to result in the improvement of patients with SAH [60], but dual antiplatelet therapy has shown promise [61]. 

### 5.3. Recombinant ADAMTS13

Alternatively, recombinant ADAMTS13 could be given to patients to assist in the degradation of ULvWF. Recombinant ADAMTS13 would allow a normalization of the abnormal vWF:ADAMTS13 ratio in patients with COVID-19. The use of rADAMTS13 could be used adjunctively with other therapies, with a potential combined approach of rADAMTS13 given alongside GPIb antagonists, GPIIbIIIa antagonists, or IV NAC. In patients with ruptured aneurysms, rADAMTS13 could be started before definitive aneurysm protection surgery with additional antiplatelet treatments delivered afterward. 

### 5.4. Plasma Exchange

Plasma exchange represents a further treatment option that we believe is more suitable for patients with COVID-19. Furthermore, rather than the original theory of using convalescent plasma to provide antibodies against the virus, plasma may, in fact, work by providing replacement ADAMTS13. As with other treatment options, early detection of patients with the most deranged vWF:ADAMTS13 ratio is essential in determining who would benefit the most from this treatment.

## 6. Conclusions

There is evidence that both COVID-19 and SAH result in endotheliopathy and the release of ULvWF, microthrombosis, and ischemia. Targeting this pathway with drugs such as GPIb antagonists and intravenous NAC or restoring the hemostatic balance using infusions of rADAMTS13 represent promising targets for both diseases.

## Figures and Tables

**Table 1:** Summary of the existing data analysing the vWF-ADAMTS13 axis in both conditions.

	COVID-19	SAH
Raised vWF levels	Y	Y
Raised ULvWF levels	Y	Not investigated
Abnormal ADAMTS13 level	Y	Y
Evidence of microthrombosis	Y	Y
